# Global Longitudinal Strain or Left Ventricular Twist and Torsion? Which Correlates Best with Ejection Fraction?

**DOI:** 10.5935/abc.20170085

**Published:** 2017-07

**Authors:** Marcio Silva Miguel Lima, Hector R Villarraga, Maria Cristina Donadio Abduch, Marta Fernandes Lima, Cecilia Beatriz Bittencourt Viana Cruz, João Cesar Nunes Sbano, Mariana Callil Voos, Wilson Mathias Junior, Jeane Mike Tsutsui

**Affiliations:** 1 Instituto do Coração (InCor), Faculdade de Medicina da Universidade de São Paulo, São Paulo, SP - Brazil; 2 Mayo Clinic, Rochester - EUA

**Keywords:** Stroke Volume, Torsion,Mechanical, Strain, Torsion Abnormality, Echocardiography,Doppler, Ventricular Dysfunction, Left

## Abstract

**Background:**

Estimative of left ventricular ejection fraction (LVEF) is a major indication for echocardiography. Speckle tracking echocardiography (STE) allows analysis of LV contraction mechanics which includes global longitudinal strain (GLS) and twist/torsion, both the most widely used. Direct comparison of correlations between these novel parameters and LVEF has never been done before.

**Objective:**

This study aims to check which one has the highest correlation with LVEF.

**Methods:**

Patients with normal LVEF (> 0,55) and systolic dysfunction (LVEF <0,55) were prospectively enrolled, and underwent echocardiogram with STE analysis. Correlation of variables was performed by linear regression analysis. In addition, correlation among levels of LV systolic impairment was also tested.

**Results:**

A total of 131 patients were included (mean age, 46 ± 14y; 43%, men). LVEF and GLS showed a strong correlation (r = 0.95; r^2^ = 0.89; p < 0.001), more evident in groups with LV systolic dysfunction than those with preserved LVEF. Good correlation was also found with global longitudinal strain rate (r = 0.85; r^2^ = 0.73; p < 0.001). Comparing to GLS, correlation of LVEF and torsional mechanics was weaker: twist (r = 0.78; r^2^ = 0.60; p < 0.001); torsion (r = 0.75; r^2^ = 0.56; p < 0.001).

**Conclusion:**

GLS of the left ventricle have highly strong positive correlation with the classical parameter of ejection fraction, especially in cases with LV systolic impairment. Longitudinal strain rate also demonstrated a good correlation. GLS increments analysis of LV systolic function. On the other hand, although being a cornerstone of LV mechanics, twist and torsion have a weaker correlation with LV ejection, comparing to GLS.

## Introduction

Left ventricle ejection fraction (LVEF) estimation is the major aim of an echocardiographic study and it is usually performed through Teichholz formula or by Simpson´s biplane rule. LVEF reflects myocardial contraction strength and is a longstanding recognized parameter in cardiology, important in a wide range of heart conditions.

**Figure 1:**
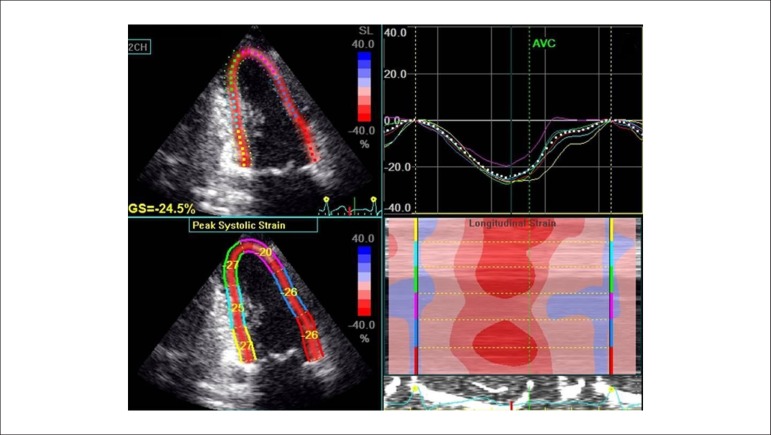
Example of global longitudinal strain analysis using speckle tracking echocardiography. GS: global longitudinal strain; AVC: aortic valve closure.

STE is a relative new method but has already been extensively validated. By tracking myocardial speckles displacement, frame-by-frame, in an angle-independent way, it allows determination of multiple aspects of LV contraction mechanics such as segmental displacement and velocity, strain and strain rate, rotations, twist/torsion, and its derivatives. Integration of all these parameters comprises a very accurate and sensitive method, which fully characterizes LV systolic function.^1-3^ This comprehensive analysis comprises determination of segmental displacement and velocity of wall motion, strain and strain rate, segmental rotations, twist/torsion, and their derivatives. Among all these parameters, global longitudinal strain (GLS) and twist/torsion are currently the most widely used (Figure 1).Torsional dynamics is the essence of LV contraction mechanics.^4-10^ Direct comparison of correlations between these novel parameters and LVEF has never been done before. Clinical usefulness of this data rely especially on cases of borderline lower values of LV ejection fraction (0,50-0,55), were exists a possibility of a systolic ventricular dysfunction. This information is crucial and has a major role on patient treatment and prognosis.

In this study we sought to correlate these newer parameters of LV systolic evaluation with LVEF in order to determine which one has the highest correlation with this classical index in echocardiography.

## Methods

### Study participants

From January 2010 to August 2013, 135 patients were prospectively recruited to participate in this single center study. Normal volunteers and patients from a general cardiologic outpatient clinic were included. Enrolment of patients comprised all range of LVEF, from normal to severe systolic impairment. Exclusion criteria were the presence of supraventricular arrhythmias (atrial fibrillation or flutter), systemic blood pressure over 180/110 mmHg, history of myocardial infarction or coronary artery disease, pacemaker, significant thyroid disease, end-stage renal failure and patients younger than 18 years-old.

The institutional review board approved the study, and all participants gave informed consent. All clinical investigations were conducted according to the principles expressed in the Declaration of Helsinki.

### Echocardiography and STE imaging acquisition

Echocardiography was performed on commercially available echocardiographic platforms equipped with MS5 probe (GE Vivid 7 and E9, GE Healthcare, Milwaukee, Wis). Comprehensive 2D-Echocardiogram and Doppler evaluation was performed following the recommendations of the American Society of Echocardiography.^11^ LVEF was measured by Simpson´s rule. Diastolic function was evaluated by mitral inflow E/A pattern and annular tissue Doppler curves (e´/a´). Valves were assessed by color, pulsed and continuous Doppler.

The echo-STE protocol included acquisition of short axis and apical views. Parasternal short-axis views were obtained at the LV base (mitral valve level) and at the LV apex, close to apical obliteration when there is still a clear visualization of segments. For this apical "cut", in order to avoid quantification bias, we created another new criterion: a clear visual identification of the apex counterclockwise rotation.

**Figure 2:**
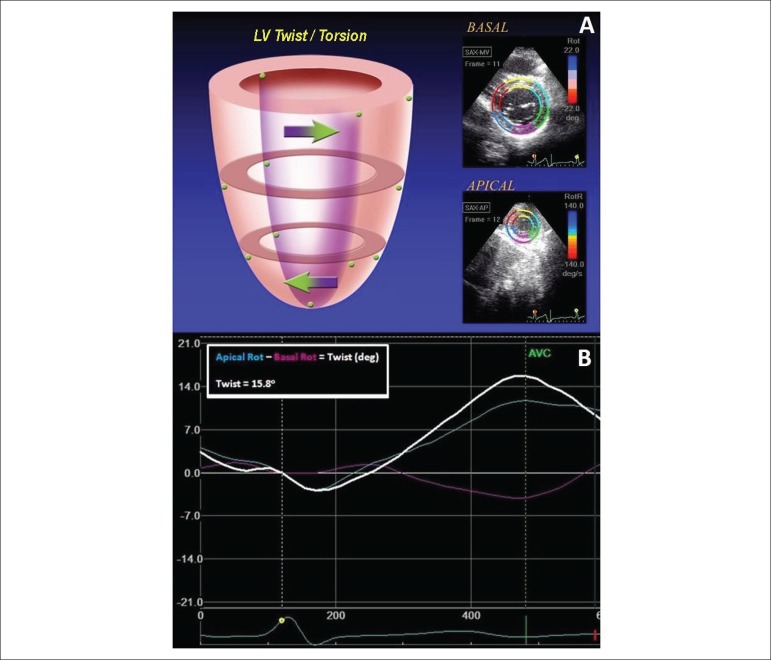
A: Representation of LV twist/torsion - clockwise rotation at the base and counterclockwise at the apex (view from the apex). B: Example of LV twist analysis (white line, LV twist; cyan line, apical rotation; pink line, basal rotation).

Left ventricular twist is the wringing motion of heart around its long axis. It is calculated as the net absolute difference between apical and basal rotations (LV_twist_ = ROT_apical_ - ROT_basal_). Torsion is a normalization of LV twist to the length of LV long axis (LV_twist_/LV_lenth_). By widely assumed convention, apical rotation had positive values and basal, negative (Figure 2).^12^

Acquisition of apical views (A3C, A2C and A4C) followed transversal images. Images were acquired at a frame-rate of 40-80 fps. Three consecutive heart cycles were stored.

Speckle tracking analysis was performed offline using a dedicated software (EchoPAC, v. BT10, GE Heathcare). For short axis images^7-12^ and for apical 3 anchor points were placed. The software automatically defined the region of interest (ROI) for the entire myocardial layer, which was divided in six color-coded segments (total: 18 segments). Careful attention was especially given to not include myocardial trabecullaes and the pericardium. Adjustments were possible. Following this step, an automatic tracking of myocardial speckles were performed and final results on the quality of this tracking were given for each color-coded segment. If there was a suboptimal tracking of one segment, adjustment was also possible. After accepting this analysis, curves were given for all variables studied and this data exported to a spreadsheet. Global values were defined as the average of segments analyzed.

Analyzes of correlation was performed using global data and by groups, according to their LVEF: group 1 (LVEF > 0.55), group 2 (LVEF: 0.55-0.30) and group 3 (LVEF < 0.30).

### Statistical analysis

Continuous variables are presented as mean ± SD, and categorical variables as numbers and proportions. Kolmogorov-Smirnov test and a histogram analysis were performed to check normality of data distribution. Variables analyzed were assumed to have a normal distribution. Correlation of variables was performed by linear regression analysis with determination of Pearson´s correlation coefficient. Six patients were randomly chosen, three with normal LVEF and three with systolic dysfunction, for analysis of interobserver and intraobserver variability. Two-tailed p values < 0.05 in a confidence interval of 95% were considered statistically significant. Statistics was performed using SPSS 20.0 for Macintosh (SPSS Inc., Chicago, IL).

## Results

Among the 135 initially patients enrolled for this study, 4 were excluded because STE analysis was not possible due to poor acoustic images. Therefore, final study population was represented by a total of 131 subjects. The overall feasibility for STE analysis was 97%. Mean age was 46 ± 14 y and 57 (43%) patients were men. A total of 27 (20.6%) individuals had hypertension.

Clinical baseline characteristics are described in table 1. The larger amount of patients was in class I (NYHA) of congestive heart failure functional classification and among all cardiovascular medication routinely prescribed, angiotensin converting enzyme inhibitor, β-blocker and diuretics were the most in use.

**Table 1:** Clinical, demographic and hemodynamic characteristics

Variables	
Age (y)	46 ± 14
Gender M	57 (43%)
Weight (Kg)	70.3 ± 14.4
Height (cm)	165 ± 10
BS (m^2^)	1.77 ± 0.21
BMI (kg/m^2^)	25.6 ± 3.9
SAH	27 (21%)
DM	6 (5%)
**CHF (NYHA) (NYHA)†**	
I	38 (29%)
II	20 (15%)
III/IV	3 (2%)
**Therapy**	
Digital	10 (8%)
ACEi	46 (35%)
βblock	50 (38%)
ARB	14 (11%)
Diuretics	40 (30%)
Aldost. Ant.	31 (24%)
HR (bpm)	69 ± 12
SBP (mmHg)	123 ± 15
DBP (mmHg)	75 ± 11

Continuous variables expressed as mean ± SD. Categorical variables expressed as frequency (proportion). BS: body surface; BMI: body mass index; SAH: systemic arterial hypertension; DM: diabetes mellitus; CHF (NYHA): functional class of congestive heart failure; ACEi: angiotensin converting enzyme inhibitor; β block: beta blocker, ARB: angiotensin II receptor blocker; Ca ++ block: calcium channel blocker; Aldost Ant: aldosterone antagonist; HR: heart rate; SBP: systolic blood pressure; DBP: diastolic blood pressure.

Conventional echocardiographic features and data from STE analyzes are shown in table 2. Mean LVEF was 0.52 ± 0.17, ranging from 0.12 to 0.72. Mean values and ranges from STE data are as follow: GLS, 17.64% ± 5.73 (3.47 -26.46); GLSRs, 1.00 s^1^ ± 0.27 (0.39 - 1.58); Twist, 14.90º ± 7.08 (-9.54 - 31.60); Torsion, 1.78º/cm ± 0.91 (-1.03 - 4.05).

**Table 2:** Echocardiographic variables

Variables	
LA (mm)	37.3 ± 6.2
LVDD (mm)	54.7 ± 10.7
LVSD (mm)	40.8 ± 13.8
LVFS (%)	26.7 ± 10.8
LVEDV (ml)	138.9 ± 66.3
LVESV (ml)	76.2 ± 61.2
LVEF (%)	51.7 ± 17.2
**Diastolic Dysfunction**	
Normal	71 (54%)
Grade I	40 (30%)
Grade II	14 (11%)
Grade III	1 (1%)
Grade IV	5 (4%)
E wave (m/s)	0.77 ± 0.21
EDT (ms)	214.0 ± 65.9
A wave (m/s)	0.60 ± 0.21
s' (cm/s)	0.06 ± 0.02
e' (cm/s)	0.08 ± 0.03
a' (cm/s)	0.07 ± 0.02
E/e'	12.7 ± 8.3
**MR Grade**	
Absent/Trivial	77 (59%)
Mild	38 (29%)
Moderate	10 (8%)
Severe	6 (5%)
GLS (%)	-17.64 (± 5.73)
GLSR (1/s)	-1.00 (± 0.27)
Twist (^o^)	14.91 (± 7.08)
Torsion (^o^/cm)	1.78 (± 0.91)

Continuous variables expressed as mean ± SD. Categorical variables expressed as frequency (proportion). LA: left atrium; LVDD: left ventricular diastolic diameter; LVSD: left ventricular systolic diameter; LVFS: left ventricular fractional shortening; LVEDV: left ventricle end diastolic volume; LVESV: left ventricle end-systolic volume; LVEF: left ventricular ejection fraction; E wave: E wave velocity, EDT: E wave deceleration time; A wave: A wave velocity; s': s' wave velocity; e‘: e' wave velocity; a': a' wave velocity; MR degree: degree of mitral regurgitation.

**Figure 3:**
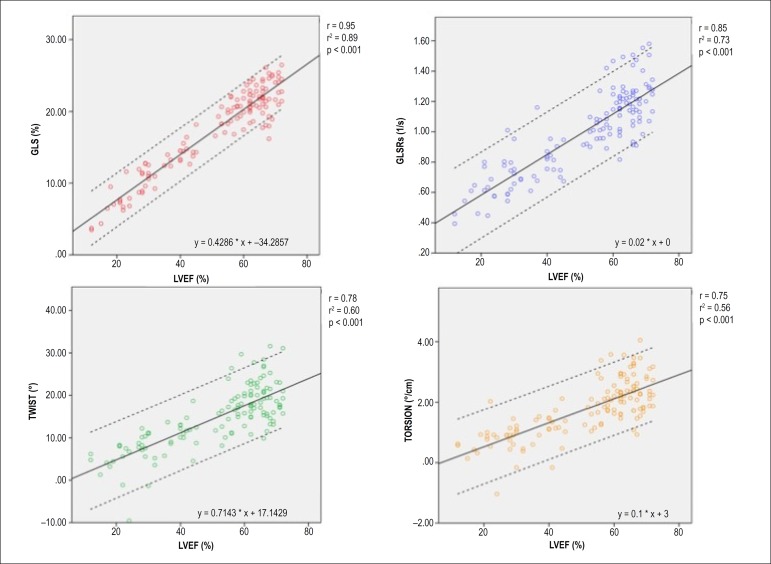
Correlation of different LV contraction parameters with LVEF (p < 0.001 for all correlations). GLS and GLSRs are displayed in absolute values. LVEF: left ventricle ejection fraction; GLS: global longitudinal strain; GLSRs: systolic global longitudinal strain rate.

A very strong correlation was identified between LVEF and GLS (r = 0.95; r^2^ = 0.89; p < 0.001) (Figure 3). Correlation between LVEF and GLSRs was also good (r = 0.85; r^2^ = 0.73; p < 0.001). On the other hand, comparing to these longitudinal parameters, correlation of LVEF and torsional mechanics was weaker: twist (r = 0.78; r^2^ = 0.60; p < 0.001); torsion (r = 0.75; r^2^ = 0.56; p < 0.001).

Analyzes of correlations according to levels of systolic impairment data is provided in table 3. Correlation was stronger between GLS and LVEF in groups with systolic mild/moderate dysfunction (r = - 0.88; p < 0.001) and severe (r = - 0.82; p < 0.001). On the other hand, this correlation was very weak in cases with preserved LV contraction (r = 0.40; p < 0.001).

**Table 3:** Correlation between LVEF and parameters of LV contraction mechanics, according to levels of systolic impairment. Pearson's coefficient (r)

	LVEF > 0.55	LVEF 0.55 – 0.30	LVEF < 0.30
LVEF X GLS	– 0.40 *	– 0.88 *	– 0.82 *
LVEF X GLSRs	– 0.36^Δ^	– 0.57^¥^	– 0.55^Φ^
LVEF X Twist	0.13^Φ^	0.44^¥^	0.34^Φ^
LVEF X Torsion	0.14^Φ^	0.45^¥^	0.23^Φ^

LVEF: Left ventricle ejection fraction; GLS: Global longitudinal strain; GLSRs: Systolic longitudinal strain rate.

* p < 0.001;

Δ p = 0.02;

¥ p = 0.03;

Φ p = NS

### Intraobserver and interobserver variabilities

Interobserver and intraobserver variabilities for longitudinal parameters were 6%, and 5%, respectively, with lower variability for longitudinal strain (3 and 4%).

For the variables obtained from short axis view, including twist and torsion, interobserver variability was 23%. Torsion had the highest (38%). Intraobserver variability was 19%. Basal rotation had the highest (32%).

## Discussion

In this study we sought to correlate these newer parameters of systolic evaluation with LVEF, in order to determine which one has the highest correlation with this classical index in echocardiography. Our major interest was in GLS and LV twist/torsion correlations, as they are the most used.

Our results showed a very strong correlation between LVEF and GLS. Such correlation has already been demonstrated experimentally by Weideman et al.^13^ and in previous clinical studies by Reant et al.,^14^ Hayat et al.^15^ and Kleijn et al.^16^ These authors also found this good correlation, especially with global area strain measurement using tridimensional speckle tracking echocardiography (r = 0.81-0.91). Goo-Yeong Cho et al.^17^ tested GLS and circumferential strain as surrogates of LVEF as prognostic tool for cardiac adverse events in patients with acute heart failure. Both of them were independent prognostic predictors of death and readmission for heart failure.^17^

Our aim was also to seek correlation with other parameters of LV torsional mechanics, twist and torsion. We also demonstrated a good correlation with LVEF, but not as strong as we found with GLS. An explanation for this fact may reside on tridimensional motion of myocardial segments. As 2D-STE misses one orientation of this movement, accuracy of tracking myocardial speckle decreases, possibly affecting these values. This is more significant on LV short-axis, where circumferential and radial measurements are made. Out-of-plane longitudinal movement is missed and has a reasonable impact on tracking, sometimes appearing as noise. On the other hand, LV circumferential and rotation movement does not have a substantial impact on longitudinal axis slightly affecting the tracking.^18^

### Clinical aspects

Results raised from this study have a clinical and practical significance, especially in cases of LVEF estimated in its lower normal limits (LVEF 0,50-0,55). In such cases, GLS may help to objectively define LV contraction strength. Lower values of GLS in a setting of a normal LVEF may represent an ejection fraction overestimation or a possible decrease in myocardial deformation, a step just before a future global LV contraction reduction. In addition, GLS analysis is relatively easy to be performed, taking only a few minutes during a conventional echocardiogram and adds a sensitive and objective parameter to left ventricle systolic function evaluation.

Finally, despite having a worse correlation with LVEF, LV twist and torsion are still good sensitive parameters that can add an objective characterization of myocardial global systolic function.

### Limitations

Notwithstanding the fact that STE method was extensively validated, it is an evolving technique, and improvements, such as on tracking accuracy, are still needed. Additionally, this accuracy is also highly dependent on image quality. Suboptimal resolution can produce a negative impact on final results.

In this study, we used 2D-STE precluding the analysis of tridimensional myocardial segments movement. The lack of analysis of one out plane movement may have had some impact on final result. Currently, 3D-STE may overcome this drawback.^19^

The subjectivity of echocardiography can bring biases of quantification. This is exemplified when referring to the "cutting" level of the LV in its short axis. Anatomical landmarks were followed to try to standardize levels, such as mitral valve to the basal level and the papillary muscles to the medium level. However, for the apical segment, there is no anatomical marker and small variations on the level of image acquisition can lead to distorted values. In order to preclude this fact, we set another new criterion: a visual identification of, at least, a tendency of rotation of the apex (differentiating from LV middle level).

## Conclusions

GLS of the left ventricle have highly strong positive correlation with the classical parameter of ejection fraction, especially in cases with LV systolic impairment. Longitudinal strain rate also demonstrated a good correlation. Clinical usefulness of this data rely especially on cases of borderline lower values of LV ejection fraction (0,50-0,55), were exists a possibility of a systolic ventricular dysfunction. GLS increments analysis of LV systolic function. On the other hand, although being a cornerstone of LV mechanics, twist and torsion have a weaker correlation with LV ejection, comparing to GLS.
